# Cap ‘n’ Collar C and Aryl Hydrocarbon Receptor Nuclear Translocator Facilitate the Expression of Glutathione S-Transferases Conferring Adaptation to Tannic Acid and Quercetin in *Micromelalopha troglodyta* (Graeser) (Lepidoptera: Notodontidae)

**DOI:** 10.3390/ijms24032190

**Published:** 2023-01-22

**Authors:** Zhiqiang Wang, Xiaoling Shi, Yujingyun Zhou, Fang Tang, Xiwu Gao, Pei Liang

**Affiliations:** 1 Co-Innovation Center for Sustainable Forestry in Southern China, Nanjing Forestry University, Nanjing 210037, China; 2 College of Forestry, Nanjing Forestry University, Nanjing 210037, China; 3 College of Plant Protection, China Agricultural University, Beijing 100193, China

**Keywords:** *Micromelalopha troglodyta* (Graeser), transcription factors, detoxification genes, promoters, plant secondary metabolites

## Abstract

*Micromelalopha troglodyta* (Graeser) (Lepidoptera: Notodontidae) is a notorious pest of poplar. Coevolution with poplars rich in plant secondary metabolites prompts *M. troglodyta* to expand effective detoxification mechanisms against toxic plant secondary metabolites. Although glutathione S-transferases (GSTs) play an important role in xenobiotic detoxification in *M. troglodyta*, it is unclear how GSTs act in response to toxic secondary metabolites in poplar. In this study, five *GST* gene core promoters were accurately identified by a 5’ loss luciferase reporter assay, and the core promoters were significantly induced by two plant secondary metabolites in vitro. Two transcription factors, cap ‘n’ collar C (CncC) and aryl hydrocarbon receptor nuclear translocator (ARNT), were cloned in *M. troglodyta*. MtCncC and MtARNT clustered well with other insect CncCs and ARNTs, respectively. In addition, MtCncC and MtARNT could bind the *MtGSTt1* promoter and strongly improve transcriptional activity, respectively. However, MtCncC and MtARNT had no regulatory function on the *MtGSTz1* promoter. Our findings revealed the molecular mechanisms of the transcription factors MtCncC and MtARNT in regulating the *GST* genes of *M. troglodyta*. These results provide useful information for the control of *M. troglodyta*.

## 1. Introduction

Plants are the main food source for animals, while insects serve as important consumers of plants. They constantly fight against each other. From a plant perspective, many plants produce defensive toxins or inhibitors to repel insects, including secondary metabolites such as isoflavones, furanocoumarins, terpenoids, alkaloids and cyanogenic glycosides [1]. From an insect perspective, insects are protected from plant secondary metabolites mainly through increased physiological tolerance, metabolic capacity of the detoxification system, or behavioral avoidance [2]. As insects attempt to increase new host plant species, these mechanisms will continue to evolve [3].

*Micromelalopha troglodyta* (Graeser), which is mainly found in China, is an important leaf-feeding pest of poplar trees and can be widely spread causing heavy losses to the forestry industry [4,5]. Poplar secondary metabolites such as tannic acid and quercetin, as toxic natural products, have a toxic effect on *M. troglodyta* [6,7]. It is well known that the powerful detoxification metabolism mechanism of insects is an important way to overcome plant chemicals [8]. There are several enzymes involved in metabolizing heterologous substances and converting them into less toxic hydrophilic compounds. Major enzyme superfamilies include glutathione S-transferases (GSTs), cytochrome P450 monooxygenases (P450s), ATP-binding cassette (ABC) transporters, UDP-glycosyltransferases (UGTs), and carboxylesterases (CarEs) [9]. GSTs are important detoxifying enzymes in *M. troglodyta* that are involved in the metabolism of plant secondary metabolites [6,8]. Mounting evidence suggests that GSTs can detoxify secondary metabolites produced by plants during the insect feeding process [10,11]. GST activity of *Myzus persicae* could be induced by different host plants of *Brassica* species, suggesting that GSTs might play roles in host plant adaptation in *M. persicase* [12]. Multiple poplar secondary metabolites could induce the expression of *GST* genes in *Lymantria dispar*, and the adaptation of *L. dispar* to plant secondary metabolites was reduced after interference with *GST* genes [13]. Red palm weevils could excrete and degrade a variety of toxic plant secondary metabolites by upregulating *GST* genes [14]. Hence, these findings demonstrated that GSTs play an important role in participating in the metabolism of xenobiotics in pests.

The detoxification genes are transcriptionally activated by a common mechanism of transcription factors [15]. Transcription factors are a class of DNA-binding proteins, including *cis*- and *trans*-acting factors, that can enhance or repress the initiation of gene transcription by binding to specific promoter sequences [16]. Cap ‘n’ collar C (CncC) and aryl hydrocarbon receptor nuclear translocator (ARNT) are two important transcription factors in insects, with an essential role in the regulation of detoxification enzymes [15,16,17,18]. Mammalian nuclear factor erythroid 2-related Factor 2 (Nrf2), a homolog of CncC, is able to induce the expression of detoxification genes in response to stress from xenobiotic substances [19,20]. CncC regulates the expression of many detoxification genes (GSTs, P450s and esterases) involved in insect metabolism and detoxification [20,21]. *Spodoptera litura* Nrf2 acted as a cis-regulatory element that activated the excretion and degradation of indole-3-methanol (I3C) and chlorpyrifos by upregulating *GSTe1*, which could improve the response of *S. litura* to phytochemicals and insecticides [22]. In *Leptinotarsa decemlineata*, several imidacloprid-resistant *GST* genes were regulated by the transcription factors CncC and Maf [23]. The aryl hydrocarbon receptor (AhR) and ARNT are members of the bHLH-PAS family of transcription factors. They are ligand-activated transcription factors involved in the perception and delivery of stimuli in response to endogenous and xenobiotic stress [24,25]. Enhancer sequences of phase I and phase II genes (such as P450s and GSTs) can be recognized and activated by the AhR-ARNT complex [26]. The *Spodoptera exigua* transcription factors AhR and ARNT coordinately regulated the expression of multiple GSTs conferring insecticide resistance [27]. In locusts, AhR could improve tolerance to chlorpyrifos by increasing the expression of *LmGSTd7* [28]. Combining the information above, CncC and ARNT are important transcriptional regulators of insect *GST* genes, and they play an important role in governing the expression of GSTs. However, the mode of regulation of *GST* genes by the two transcription factors in *M. troglodyta* is not clear.

We cloned five *GST* promoters of *M. troglodyta* and demonstrated that tannic acid could induce the activity of these five *MtGST* promoters [4]. To date, it has not been reported at home or abroad how the expression of *GST* in *M. troglodyta* is regulated to adapt to plant secondary metabolite stress. In this study, we focused on the following three questions: (1) Where is the core region of the *MtGST* promoter located? (2) Will the core region of the *MtGST* promoter be responsive to plant secondary metabolites? and (3) Do MtCncC and MtARNT interact with the promoters of *GST*? This study elucidated the role of MtCncC and MtARNT in regulating GST metabolism of plant secondary metabolites, which would be helpful to find new target genes to control *M. troglodyta*.

## 2. Results

### 2.1. MtGST Promoter Activity Analysis

**Figure 1:**
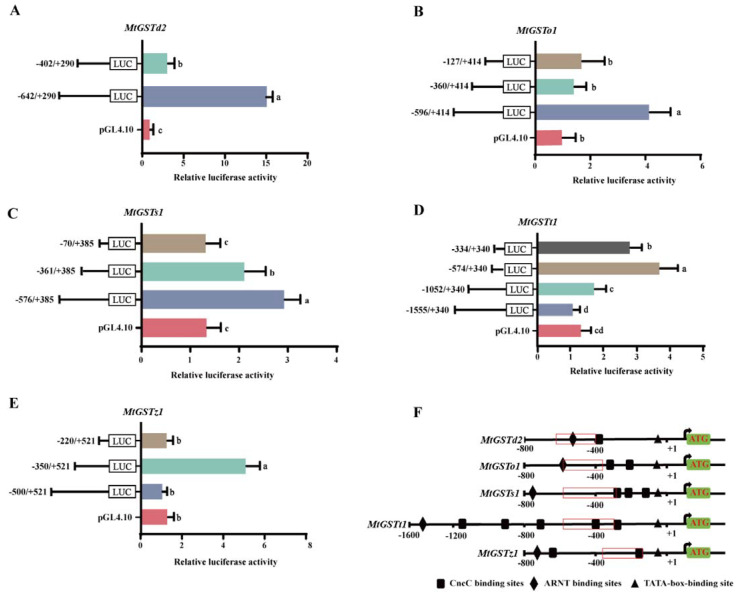
Relative luciferase activities of the 5’ loss fragment of *MtGST* promoters. (**A**): *MtGSTd2* promoter constructs; (**B**): *MtGSTo1* promoter constructs; (**C**): *MtGSTs1* promoter constructs; (**D**): *MtGSTt1* promoter constructs; (**E**): *MtGSTz1* promoter constructs; F: Prediction analysis of the promoter regions of five *MtGST* genes. Each value is presented as the mean ± SD of three replicates, and different lowercase letters show significant differences (*p* < 0.05). (**F**): The core regions deduced from five *MtGST* promoters. The location of nucleotides was marked relative to the transcription start site indicated by +1 and the translation start site indicated by ATG. The red box represents the core region of each promoter.

In our previous study, we cloned five *MtGST* promoters. To determine the regions that are essential for *GST* expression, we constructed several deletion structures containing 5’ loss fragments and inserted them into the pGL4.10-Basic vector (Figure 1).

The pGL4.10-(−642/+290) vector of *MtGSTd2* promoter had relatively high luciferase expression compared with that of pGL4.10-(−402/+290), which showed that sequences were essential for the transcription of *MtGSTd2* located in the 240 bp region between −642 and −402 bp (Figure 1A). The pGL4.10-(−596/+414) vector of *MtGSTo1* promoter exhibited relatively high luciferase expression compared with that of pGL4.10-(−360/+414) and pGL4.10-(−127/+414), which suggested that −596 to −360 region was critical for *MtGSTo1* transcription (Figure 1B). The pGL4.10-(−576/+385) vector of *MtGSTs1* promoter displayed relatively high luciferase expression than that of the pGL4.10-(−361/+385), and pGL4.10-(−70/+385) of *MtGSTs1* had low luciferase expression compared to pGL4.10-(−361/+385), which suggested that sequences were essential for the transcription of *MtGSTs1* located in the 215 bp region between −576 and −361 bp (Figure 1C). The pGL4.10-(−574/+340) vector of *MtGSTt1* promoter had relatively high luciferase expression compared with that of pGL4.10-(−334/+340), and the pGL4.10-(−334/+340) vector of *MtGSTt1* had high luciferase expression levels compared to pGL4.10-(−1052/+340) and pGL4.10-(−1555/+340), which showed that the 240 bp transcription region between −574 and −334 was critical for *MtGSTt1* expression (Figure 1D). The pGL4.10-(−350/+521) vector of *MtGSTz1* promoter had relatively high luciferase expression compared with that of pGL4.10-(−500/+521) and pGL4.10-(−220/+521), which suggested that sequences between −350 and −220 were critical for *MtGSTz1* expression (Figure 1E). The main regulatory regions of the five *MtGST* promoters were shown in Figure 1F.

### 2.2. Induction Effect of Tannic Acid and Quercetin on the Core Region of Two Promoters

**Figure 2:**
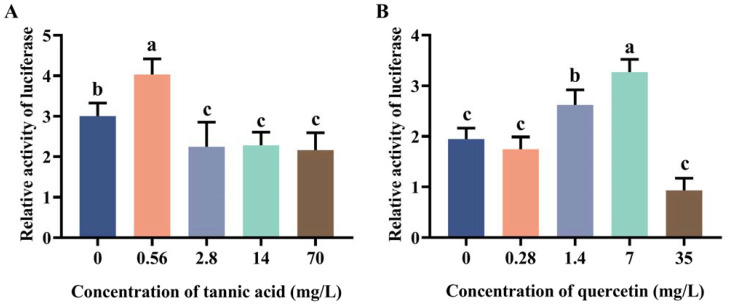
Promoter activity of *MtGSTt1* (−574/+340) under tannic acid (**A**) and quercetin (**B**) stress. Each value is presented as the mean ± SD of three replicates, and different lowercase letters show significant differences (*p* < 0.05).

**Figure 3:**
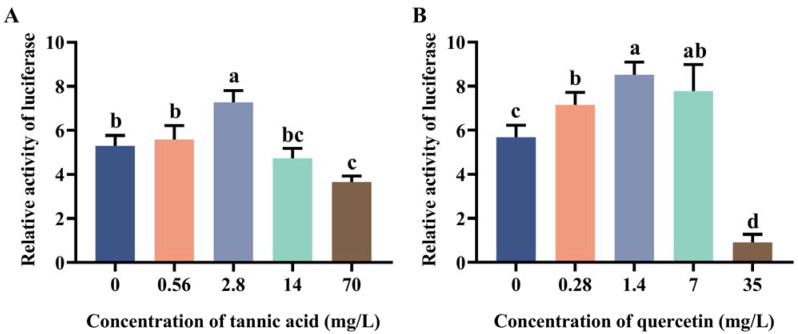
Promoter activity of *MtGSTz1* (−350/+521) under tannic acid (**A**) and quercetin (**B**) stress. Each value is presented as the mean ± SD of three replicates, and different lowercase letters show significant differences (*p* < 0.05).

Due to the high transcriptional activity of *MtGSTt1* (−574/+340) and *MtGSTz1* (−350/+521) promoters, we used them as examples to explore the promoter response to tannic acid and quercetin (Figure 2 and Figure 3). The promoter activity of *MtGSTt1* (−574/+340) was significantly induced by tannic acid at a low concentration (0.56 mg/L), while it was inhibited by tannic acid at 2.8, 14 and 70 mg/L (Figure 2A). The *MtGSTt1* (−574/+340) promoter showed an increasing and then decreasing trend under quercetin stress, and the highest promoter activity was observed when the quercetin concentration was 7 mg/L (Figure 2B). These data showed that the promoter activity of the core region of *MtGSTt1* (−574/+340) could be induced by tannic acid and quercetin. For *MtGSTz1* (−350/+521) promoter, the promoter activity was strongly induced by 2.8 mg/L tannic acid (Figure 3A), while it was repressed at 70 mg/L tannic acid (Figure 3A). We also observed that the promoter activity of *MtGSTz1* (−350/+521) was notably increased when Sf9 cells were treated with quercetin at 0.28, 1.4 and 7 mg/L and sharply repressed at 35 mg/L (Figure 3B). These data suggested that tannic acid and quercetin could influence the core region of *MtGSTz1* promoter.

### 2.3. Cloning and Phylogenetic Analysis of MtCncC

**Figure 4:**
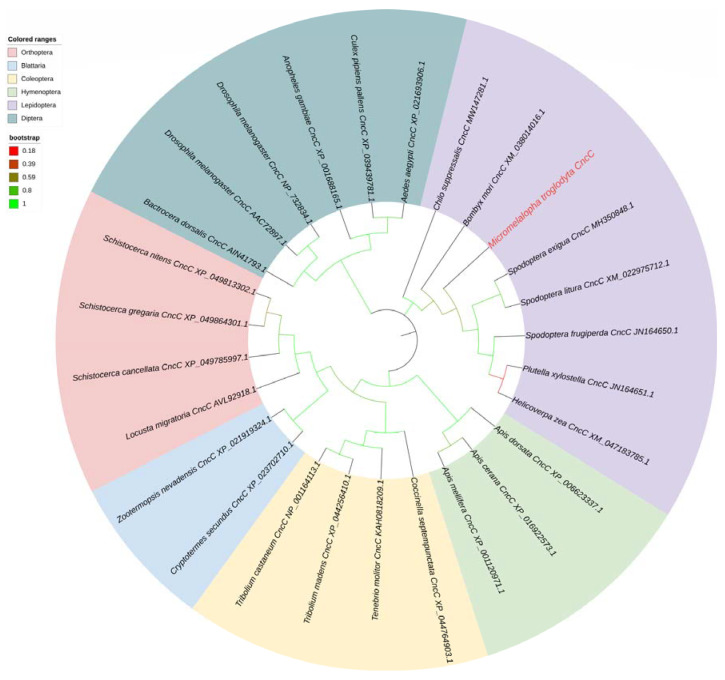
Phylogenetic analysis of the CncC sequences of several insect species. The CncC gene name was shown as the Latin name of the species, CncC and the NCBI gene accession number, and the *M. troglodyta* CncC was marked in red and bold. The branches for Lepidoptera, Diptera, Blattodea, Coleoptera, Hymenoptera, and Orthoptera CncCs were shaded in different colors, and branch colors represented bootstrap values.

To explore the regulatory mode of promoters by transcription factors. The *CncC* gene of *M. troglodyta* was obtained from the transcriptome database and confirmed by cloning and resequencing. *MtCncC* is a 1671 bp open reading frame (ORF) sequence and encodes 556 AA residues. The amino acid sequence of *MtCncC* is listed in Supplemental Material S1. Twenty-six representative insect CncC amino acid sequences were selected for constructing the phylogenetic tree using the maximum likelihood (ML) method of MEGA X based on the multiple alignment built with Clustal W. The phylogenetic analysis showed that CncCs from different species were classified into six order clusters, and MtCncC was clearly classified into Lepidoptera subclusters, which suggested that MtCncC had a high identity with other lepidopteran insects CncCs (Figure 4).

### 2.4. Cloning and Phylogenetic Analysis of MtARNT

**Figure 5:**
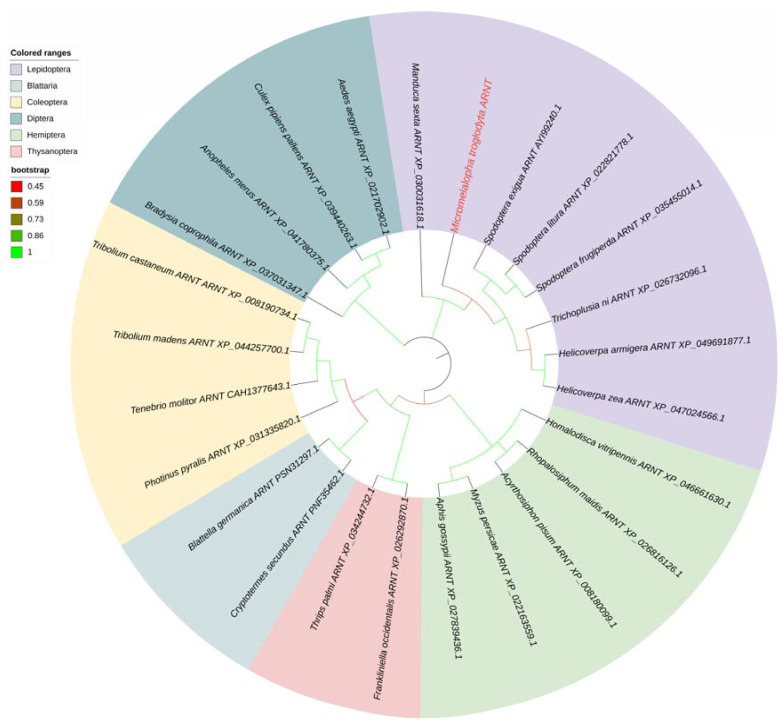
Phylogenetic analysis of the ARNT sequences of several insect species. The ARNT gene name was shown as the Latin name of the species, ARNT and the NCBI gene accession number, and the *M. troglodyta* ARNT was marked in red and bold. The branches for Lepidoptera, Diptera, Blattodea, Coleoptera, Hymenoptera, and Thysanoptera ARNTs were shaded in different colors, and branch colors represented bootstrap values.

Another transcription factor *ARNT* was also identified from the transcriptome database and confirmed by cloning and resequencing. *MtARNT* is a 1440 bp ORF sequence and encodes 479 AA residues. The amino acid sequence of *MtARNT* is listed in Supplemental Material S1. The phylogenetic tree of MtARNT was built according to the above method. Phylogenetic analysis showed that MtARNT was grouped into Lepidoptera subclusters and had a high similarity with ARNTs from other insects (Figure 5).

### 2.5. Analysis of the Transcriptional Activity of MtGST Promoters Regulated by MtCncC/MtARNT

**Figure 6:**
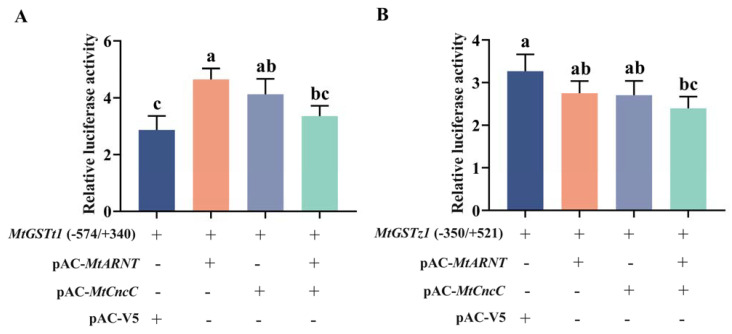
Relative luciferase activity in cells transfected with the *MtGST* promoters, MtCncC and MtARNT. (**A**) *MtGSTt1* (−574/+340) promoter; (**B**) *MtGSTz1* (−350/+521) promoter. Each promoter activity was measured in the absence of protein (promoter + empty pAC-V5 basic vector), in the presence of a protein (MtARNT or MtCncC) or in the presence of two proteins (MtCncC and MtARNT). Each value is presented as the mean ± SD of three replicates, and lowercase letters show significant differences (*p* < 0.05).

To further determine the interaction relationship between the MtCncC/MtARNT and *MtGST* promoters, we selected the *MtGSTt1* (−574/+340) and *MtGSTz1* (−350/+521) promoters with high transcription activity to verify the regulatory role of ARNT and CncC. pGL4.10-*MtGSTt1* (−574/+340) and pGL4.10-*MtGSTz1* (−350/+521) promoters were transfected into Sf9 cells. *MtCncC* and *MtARNT* sequences were constructed into pAC-V5 basic expression vector and named pAC-*CncC* and pAC-*ARNT*, respectively. The pAC-V5 basic expression vector was used as a negative control. The transcription factors MtCncC/MtARNT and the promoter constructs were cotransfected into Sf9 cells. As shown in Figure 6A, the luciferase activity of pGL4.10-*MtGSTt1* (−574/+340) promoter strongly increased when cotransfected with pAC-*CncC* or pAC-*ARNT*. These results showed that the expression of *MtCncC*/*MtARNT* in Sf9 cells facilitates *MtGSTt1* (−574/+340) promoter transcription, but there was no significant change when cotransfected with pAC-*CncC* and pAC-*ARNT*. When pAC-*CncC*/pAC-*ARNT* was cotransfected with pGL4.10-*MtGSTz1* (−350/+521) promoter, the transcriptional activity of the *MtGSTz1* (−350/+521) promoter was not increased (Figure 6B).

## 3. Discussion

GSTs are broadly distributed and important detoxifying enzymes in aerobic organisms that catalyze glutathione (GSH) binding to endogenous and exogenous compounds and excrete them outside cells, thus reducing their damage to the cells [29,30]. In insects, GSTs are a family of multifunctional enzymes involved in the detoxification of toxic compounds, including plant secondary metabolites [4,31,32]. There are six cytoplasmic *GST* gene families, including epsilon, omega, delta, theta, sigma, and zeta [33]. In a previous study, we demonstrated that all five *GST* genes of *M. troglodyta* could be significantly induced by tannic acid, which belong to the omega, delta, theta, sigma, and zeta families. Subsequently, we cloned the 5’ flanking promoter sequences of these five *GST* genes and found that they could be induced by tannic acid [4].

The promoter is a very important regulatory element in gene transcription that determines the pattern and intensity of gene expression [34]. Many inducible promoters have been identified from insects, plants and pathogens to explore the in-depth mechanisms of their regulation [35,36,37]. The promoter of *Drosophila* heat shock protein (*Hsp70*) could enhance the expression of *Hsp70* more than 200-fold after heat stimulation treatment [36]. Adding a stress-inducible promoter before the *DREB1A* gene in plants could enhance the drought, high salt and low temperature resistance of transgenic plants [37]. *Bombyx mori* nucleopolyhedrovirus (BmNPV)-inducible promoters were applied for gene therapy [38]. Currently, the promoters of *GST* have been reported in a few insects. In *S. litura*, the *GST* promoter acted as an important element for upregulating the expression of *GST* and improved *S. litura* tolerance to insecticides [22]. It was reported that the promoters of *S. exigua GST*s were coregulated by two transcription factors, which enhanced the resistance of insects to xenobiotic stress [27]. Although we previously obtained five *MtGST* promoters, their regulatory mechanisms for *MtGST* genes are not clear. In the present study, we further identified the core regions of the five *MtGST* promoters in vitro by a 5’ loss fragment assay. The activity of the core regions of *MtGSTt1* (−574/+340) and *MtGSTz1* (−350/+521) promoters were also well induced by low concentrations of tannic acid and quercetin, which suggested that the core sequences of *MtGST* promoters have significant activity in response to plant secondary metabolites. However, we also found that higher concentrations of tannic acid and quercetin would decrease the activity of luciferase despite that they were not toxic to Sf9 cells. Based on a previous study, when human cells were exposed to xenobiotic stress, low concentrations of xenobiotics induced the promoter activity of pituitary adenylate cyclase-activating polypeptide (PACAP) receptor 1 (PAC1-R) while high concentrations of xenobiotics inhibited *PAC1-R* promoter activity [39]. Thus, we hypothesized that the relationship between *GST* promoter activity and plant secondary metabolites (tannic acid and quercetin) also presented a dose-dependent way. Once the concentrations of plant secondary metabolites exceeded the range causing induction, they might inhibit the activity of promoters. These findings provided useful information for understanding the mechanism of *GST* transcriptional regulation in *M. troglodyta*.

The transcription factor Nrf2, which is a member of the basic leucine-zipper family, is an oxygen-sensitive transcription factor and a vital physiological stress response mechanism in organisms [40]. Under oxidative stress, Nrf2 can translocate into the nucleus to bind to antioxidant response elements (AREs) and heterodimerize with MafK to regulate the expression of detoxification genes [41]. The mutation of *Nrf2* in mice makes it more sensitive to xenobiotic stress [42]. Nrf2 can recognize specific DNA sequences in the presence of nuclear factor-erythroid 2 [43]. CncC in insects is homologous to Nrf2 and is an important transcription factor for regulating detoxification genes. In silkworm, both *CncC* and detoxification genes (including GSTs and P450s) regulated by CncC were upregulated after phoxim treatment [44]. The CncC-mediated detoxification pathway was associated with oxidative stress in *Drosophila*, and it was found that CncC could upregulate *GSTd* expression to enhance the ability to resist oxidative stress [45]. Nrf2 was able to regulate the detoxification enzyme gene *CYP6A2* and increase resistance to DTT in *Drosophila* [46]. In *Tribolium castaneum*, the transcription factors CncC and Maf could regulate the expression of the *CYP6BQ* gene and increase resistance to deltamethrin [47]. Based on the results of phylogenetic analysis in this study, MtCncC was highly similar to CncCs from other insects. Therefore, we speculated that *MtCncC* is relatively conserved and has similar characteristics to other *CncCs*. In our study, by cotransfecting constructs containing *MtCncC* sequences and *MtGST* promoter sequences, we observed a significant induction of the *MtGSTt1* (−574/+340) promoter by MtCncC, which suggests that MtCncC acts as a transcription factor responsible for the activity of the *MtGSTt1* (−574/+340) promoter.

ARNT is also a regulatory element of xenobiotic stress response genes and a member of the bHLH-PAS transcription factor superfamily [25]. AhR is another bHLH-PAS protein family member that is a ligand-activated transcription factor [48]. In vertebrates, AhR has two isoforms, AhR1 and AhR2. AhR1 is found in all vertebrates, while AhR2 is present in some vertebrates [49]. AhR and ARNT can form heterodimers to bind enhancer DNA sequences and activate antioxidant and xenobiotic metabolic genes such as GSTs and P450s [26,50]. In mammals, some *GSTs* were regulated by AhR/ARNT [51,52]. In insects, AhR/ARNT was associated with the regulation of *Aphis gossypii* Glover *CYP450* to improve its tolerance to spirotetramat [53]. In *M. persicae*, AhR/ARNT could upregulate the expression levels of *CYP450* to confer resistance to pesticides [54]. NlARNT could bind to the *CarE7* promoter and strongly induce transcriptional activity to enhance resistance to xenogenic stress in *Nilaparvata lugens* [55]. In this study, the phylogenetic relationship of MtARNT was closely related to that of other insect ARNTs. We hypothesized that MtARNT is highly similar to other ARNTs and has similar functions to other ARNTs. In this study, by cotransfecting constructs containing *MtARNT* sequences and *MtGST* promoter sequences, the *MtGSTt1* (−574/+340) promoter was significantly induced by MtARNT, which suggests that MtARNT acts as an important cis-regulatory element responsible for the transcriptional activity of *MtGSTt1* (−574/+340). CncC and ARNT coordinately regulated the expression of *GST* in *S. exigua* [27]. In mammals, the interaction between AhR and Nrf2 may be achieved through multiple mechanisms, including *Nrf2* as a target gene of AhR, indirect activation of Nrf2 via *CYP1A1-*generated reactive oxygen species, and direct cross-interaction of AhR/XRE and Nrf2/ARE signaling [56]. According to our results, MtCncC and MtARNT did not coregulate the *MtGST* promoters and even appeared to reduce the transcriptional activity of the promoters. Thus, we speculated that MtCncC and MtARNT regulate the *GST* genes of *M. troglodyta* in a complex process.

In summary, this study identified the core regions of the five *MtGST* promoters and demonstrated their involvement in the response to tannic acid and quercetin stress. Furthermore, we identified two important transcription factors MtCncC and MtARNT involved in the regulation of the *GST* gene promoter. These results suggested that transcription factors regulate the expression of *GSTs* conferring resistance to plant secondary metabolites in *M. troglodyta*, and provided useful information for a better understanding of the regulatory mechanism between transcription factors and GSTs in *M. troglodyta*. Future studies will need to examine the mechanism of posttranscriptional regulation of *GSTs* in *M. troglodyta*.

## 4. Materials and Methods

### 4.1. Insect Rearing and Cell Culture

*M. troglodyta* larvae were gathered from poplar (*Populus* × *euramericana* ‘Nanlin 895’) trees in Nanjing, Jiangsu Province, China. The larvae were fed fresh poplar leaves with a photoperiod of 16 h:8 h (light: dark), a temperature of 26 ± 1 °C and a relative humidity of 70–80%. Third-instar larvae were used for subsequent experiments. Sf9 cells were routinely maintained with SF-900 II serum-free medium (Invitrogen, Carlsbad, CA, USA) with 10% fetal bovine serum (HyClone, Logan, UT, USA), 50 mg/mL streptomycin and 50 mg/mL penicillin (Invitrogen) at 28 °C. Sf9 cells were cultured for 3 days and then used for transfection experiments.

### 4.2. Cloning and Sequencing 5’ Loss Fragments of GST Promoters

First, the transcription factor-binding sites for all full-length *MtGST* promoter sequences were predicted on the website http://alggen.lsi.upc.es (accessed on 10 November 2022) to avoid disrupting the integrity of the binding sites when the promoters of different fragments were cloned. The 5’ loss fragments of each *MtGST* promoter were amplified from the full-length sequences of the *MtGST* promoters using TaKaRa *Ex* Premier™ DNA Polymerase (Takara, Dalian, Liaoning, China). All primers were designed by Primer 5 software and were listed in Table 1. Each forward primer sequence and reverse primer sequence were added with Nhe I and Xho I restriction enzyme cleavage sites, respectively. Each 5’ loss fragment of *MtGST* promoter was ligated to a TA clone vector pMD-19T (Takara, Dalian, Liaoning, China), and the correct clone product was obtained by sequencing. The PGL4.10-Basic vector and the pMD-19T with the 5’ loss fragment were digested with Nhe I and Xho I. Then, the 5’ loss fragment of *MtGST* promoter was ligated to the PGL4.10-Basic vector using T4 DNA ligase (Takara, Dalian, Liaoning, China), and the ligation product was transformed into *E. coli* cells. The plasmid DNA was purified from *E. coli* cells for subsequent cell transfection experiments.

### 4.3. Promoter Activity Analysis by Luciferase Reporter Assays

Sf9 cells (2.0 × 10^6^ /well) were cultured in a 24-well culture plate, and each 5’ loss fragment promoter plasmid (700 ng/well) and pRL-TK (interference renilla luciferase reporter plasmid, Promega, Madison, WI, USA) (70 ng/well) were cotransfected using 2 µL/well Cellfectin II reagent (Invitrogen) in accordance with our previous method (Tang et al., 2020). After 48 h, Sf9 cells were harvested and lysed in 1 × passive lysis buffer (Promega), and the renilla and firefly luciferase activities were measured using the Dual-Luciferase^®^ Reporter Assay System kit (Promega) on an FLx800^TM^ fluorescence microplate reader (BioTek, Winooski, VT, USA). The promoter activity was calculated by normalizing the relative activity of firefly luciferase with that of renilla luciferase. Three replicates were performed for each treatment independently.

Tannic acid was initially solubilized in a small volume of acetone and then diluted in sterilized water to 70, 14, 2.8 and 0.56 mg/L, and sterilized water was used as a control. Quercetin was serially diluted in acetone to 35, 7, 1.4 and 0.28 mg/L, and acetone was used as a control. The concentrations of tannic acid and quercetin were determined according to previous study [4,57]. Sf9 cells (2.0 × 10^6^ /well) were cultured in a 24-well culture plate, and then each 5’ loss fragment promoter plasmid (700 ng/well) and pRL-TK (interference plasmid) (70 ng/well) were cotransfected using 2 µL/well Cellfectin II reagent (Invitrogen). At 5 h posttransfection, we changed the transfection solution to cell culture medium containing 10 µL of tannic acid or quercetin with serum and double antibiotics. After 48 h, we measured luciferase activity using a Dual-Luciferase^®^ Reporter Assay System kit on a microplate reader. The luciferase activity was calculated according to the above method.

### 4.4. Cloning the Sequences of MtCncC and MtARNT Genes

Total RNA was extracted from third-instar larvae using TRIzol Reagent (Takara, Dalian, Liaoning, China) according to the protocol. The quality and integrity of RNA were examined by a NanoDrop spectrophotometer and agarose gel electrophoresis, respectively. *M. troglodyta* RNA was reverse transcribed using the PrimeScript^TM^ 1st Strand cDNA Synthesis Kit (Takara, Dalian, Liaoning, China), and the cDNA was used for cloning *MtCncC* and *MtARNT*. The primers for cloning *MtCncC* and *MtARNT* were designed according to the transcriptome database and were listed in Table 1. Polymerase chain reaction (PCR) was performed using Premix *Ex* Taq™ (Takara, Dalian, Liaoning, China). The PCR program was as follows: 98 °C for 3 min; 35 cycles of 98 °C for 10 s, approximately 60 °C for 30 s and 72 °C for 90 s; an extension cycle of 72 °C for 5 min. The *MtCncC* and *MtARNT* DNA were ligated to the pMD-19T clone vector. The constructs were transformed into *E. coli* cells and sequenced by Sangon Biotech (Shanghai) Co., Ltd. The amino acid (AA) sequences of *MtCncC* and *MtARNT* were deduced from the NCBI Open Reading Frame (ORF) finder (https://www.ncbi.nlm.nih.gov/orffinder/, accessed on 10 November 2022).

### 4.5. Phylogenetic Analysis of MtCncC and MtARNT

A phylogenetic tree was constructed to investigate the relationship between MtCncC and other insect CncC, and we picked Cap ‘n’ Collar as a keyword to query the nonredundant database (https://www.ncbi.nlm.nih.gov/ (accessed on 10 November 2022)). Multiple AA sequence alignment analysis was carried out using MEGA X (version 10.1) and Clustal X software (version 2.1). The phylogenetic tree was inferred by the maximum likelihood (ML) method in MEGA X with 1000 bootstrap replicates. The phylogenetic tree of MtARNT was inferred using the same methods.

### 4.6. Cotransfection of MtCncC and MtARNT with MtGSTt1 (−574/+340) or MtGSTz1 (−350/+521) Promoter

Using two primer pairs pAC-V5-*CncC* and pAC-V5-*ARNT* (Table 1), the *MtCncC* and *MtARNT* were amplified, respectively. Then *MtCncC* and *MtARNT* were cloned into pAC-V5 (Invitrogen). Sf9 cells (2.0 × 10^6^/well) were cultured in a 24-well culture plate, and 350 ng of the promoter plasmid and 350 ng of the pAC-V5, pAC-V5-*MtARNT*, pAC-V5-*MtCncC* or pAC-V5-*MtARNT* and pAC-V5-*MtCncC* expression plasmids were cotransfected using 2 µL/well Cellfectin II reagent (Invitrogen). After 48 h induction, Sf9 cells were harvested and lysed to measure the renilla and firefly luciferase activities.

### 4.7. Statistical Analysis

ANOVA of the data collected from these experiments was performed using InStat software (GraphPad, San Diego, CA, USA). The significant differences of all two samples were evaluated using Student’s *t* test (two-tailed unpaired *t* test). The statistical significance of multisample comparisons was assessed with one-way ANOVA followed by Tukey’s multiple comparisons. A value of *p* < 0.05 was considered significantly different.

## Figures and Tables

**Table 1:** The primers used for this study.

Primer Names	Forward Primer Sequences (5’-3′)	Reverse Primer Sequences (5’-3′)	Experiments
Pro-*MtGSTd2* (−642/+290)	CTCGAGAGATTACTATAGGGCACG	GCTAGCAGGTAGTACAGGTCG	5’ loss fragment *MtGST* promoters
Pro-*MtGSTd2* (−402/+290)	CTCGAGCTTAGCTGGTTGC	GCTAGCAGGTAGTACAGGTCG	
Pro-*MtGSTo1* (−596/+414)	CTCGAGATTACTATAGGGCACGC	GCTAGCGGTTTGTAAATGTTTT	
Pro-*MtGSTo1* (−360/+414)	CTCGAGTAACAATTGGCAC	GCTAGCGAATACACGCAGTTT	
Pro-*MtGSTo1* (−127/+414)	CTCGAGTCCGACTTTGTGAA	GCTAGCGAATACACGCAGTTT	
Pro-*MtGSTs1* (−576/+385)	CTCGAGCGACGAAGGCTT	GCTAGCTCAGTAACAACGAC	
Pro-*MtGSTs1* (−361/+385)	CTCGAGCCTTCCAGTAGTTTG	GCTAGCTCAGTAACAACGAC	
Pro-*MtGSTs1* (−70/+385)	CTCGAGCATCGTTTCTAGAGT	GCTAGCTCAGTAACAACGAC	
Pro-*MtGSTt1* (−1552/+340)	CTCGAGTGCCTGCAGGTC	GCTAGCGATTTCTTCACAGAGTG	
Pro-*MtGSTt1* (−1055/+340)	CTCGAGTGTCCCGTCACA	GCTAGCGATTTCTTCACAGAGTG	
Pro-*MtGSTt1* (−574/+340)	CTCGAGTTGGACTATAGCCTTC	GCTAGCGATTTCTTCACAGAGTG	
Pro-*MtGSTt1* (−334/+340)	CTCGAGCATGCTATGCCC	GCTAGCGATTTCTTCACAGAGTG	
Pro-*MtGSTz1* (−500/+521)	CTCGAGGGCACGCGTG	GCTAGCAGCGATAGATAAGCG	
Pro-*MtGSTz1* (−350/+521)	CTCGAGGACGTTGGCATT	GCTAGCAGCGATAGATAAGCG	
Pro-*MtGSTz1* (−220/+521)	CTCGAGTCTTATTTGGAAACG	GCTAGCAGCGATAGATAAGCG	
*ARNT*	ATGAGTTTATTGACTGATGTCTGCCT	TCACCGGCGCCCGCCGCC	Gene cloning
*CncC*	ATGCTGCACCCGGCCAT	TCACTGATCGTAGTGCTTCGCTT	
pAC-V5-*ARNT*	GCGGCCGCATGAGTTTATTGACTGAT	GGCGCGCCCCGGCGCCCGCCGCC	pAC-V5 vector construct
pAC-V5-*CncC*	GCGGCCGCATGCTGCACCCGGCCAT	GGCGCGCCCTGATCGTAGTGCTTCGCTT	

Note: The underline within primers indicated the restriction sites.

## Data Availability

Data are contained within the article and Supplementary Material.

## Supplementary Materials

The following supporting information can be downloaded at: https://www.mdpi.com/article/10.3390/ijms24032190/s1.
